# Microalbuminuria and BMI

**DOI:** 10.4103/0971-4065.59345

**Published:** 2009-10

**Authors:** V. Wiwanitkit

**Affiliations:** Wiwanitkit House, Bangkhae, Bangkok - 10160, Thailand

Sir,

I read the recent publication by Chowta *et al*. with a great interest.[1] Chowta *et al.* reached the conclusion that “there is no effect of BMI and sex on the prevalence of microalbuminuria”.[1] Indeed, the correlation between BMI and microalbuminuria is widely discussed. In a recent publication by Sibal *et al.*,[2] BMI is a good predictor for macrovascular complication of DM but not for microalbuminuria. However, the strong correlation between BMI and microalbuminuria was reported in another study among patients with hypertension.[3] The correlation between microalbuminuria and BMI is still a myth that needs further complete assessment.
